# Data Fusion Techniques for the Integration of Multi-Domain Genomic Data from Uveal Melanoma

**DOI:** 10.3390/cancers11101434

**Published:** 2019-09-26

**Authors:** Max Pfeffer, André Uschmajew, Adriana Amaro, Ulrich Pfeffer

**Affiliations:** 1 Max Planck Institute for Mathematics in the Sciences, 04103 Leipzig, Germany; uschmajew@mis.mpg.de; 2 IRCCS Ospedale Policlinico San Martino, 16132 Genova, Italy; adriana.amaro82@gmail.com

**Keywords:** DNA-methylation, copy number alteration, gene expression profile, metastasis, tumor classification, tumor subtypes, data fusion, singular value decomposition, constrained matrix factorization, similarity network fusion

## Abstract

Uveal melanoma (UM) is a rare cancer that is well characterized at the molecular level. Two to four classes have been identified by the analyses of gene expression (mRNA, ncRNA), DNA copy number, DNA-methylation and somatic mutations yet no factual integration of these data has been reported. We therefore applied novel algorithms for data fusion, joint Singular Value Decomposition (jSVD) and joint Constrained Matrix Factorization (jCMF), as well as similarity network fusion (SNF), for the integration of gene expression, methylation and copy number data that we applied to the Cancer Genome Atlas (TCGA) UM dataset. Variant features that most strongly impact on definition of classes were extracted for biological interpretation of the classes. Data fusion allows for the identification of the two to four classes previously described. Not all of these classes are evident at all levels indicating that integrative analyses add to genomic discrimination power. The classes are also characterized by different frequencies of somatic mutations in putative driver genes (GNAQ, GNA11, SF3B1, BAP1). Innovative data fusion techniques confirm, as expected, the existence of two main types of uveal melanoma mainly characterized by copy number alterations. Subtypes were also confirmed but are somewhat less defined. Data fusion allows for real integration of multi-domain genomic data.

## 1. Introduction

Uveal melanoma (UM) accounts for approximately 5% of all melanomas [1]. The incidence of UM in Europe shows a characteristic increase from south to north, from <2 per million in Spain and southern Italy to >8 per million in Norway and Denmark [2]. Therapy, enucleation, endoresection or radiotherapy, almost completely controls primary tumors but 25% and 34% of UM patients develop metastases within 5 and 10 years, respectively. Median survival after diagnosis of metastatic UM is approximately one year [3]. The long-term cumulative melanoma-related mortality rate is over 50% for medium and large tumors at 25 years after primary treatment [4]. UM is clearly distinct from cutaneous melanoma by different driver mutations, different chromosomal copy number alterations, a much lower mutational burden, and thus has to be treated as a different disease despite the common developmental origin of uveal and cutaneous melanocytes [5,6].

UM shows a mean of 17 mutations in coding regions of protein coding genes [7], approximately 80% of cases show a mutation in the alpha subunit of one of the two G-protein alpha subunits, GNAQ and GNA11 [8]. Mutations in the tumor suppressor gene BAP1 [9] are associated with elevated metastatic risk and mutations in SF3B1 [10], a gene encoding a component of the splicing machinery, confer intermediate risk with retarded development of metastases. The relatively frequent mutations of the translational elongation factor gene EIF1AX [11] do apparently not influence metastatic risk. Monosomy of chromosome 3 and amplification of chr8q are associated with metastatic risk, while amplification of chr6p is associated with a reduced risk in cases with concomitant monosomy of chr3. For recent reviews see refs. [5,6,12].

Genomics has greatly contributed to the identification of prognostic classes that are distinguished by different gene expression profiles [13,14,15], chromosomal copy number alterations [16] and somatic mutations [8,9,10,17]. As a result, cytogenetics, transcriptional profiling and/or BAP1 sequencing or immunohistochemistry have been integrated in routine prognostic assessment by the pathologist. The initially two prognostic classes, mainly distinguished by chromosome 3 status, have been extended to three classes since cases with disomy of chromosome 3 and SF3B1 mutations tend to develop metastases with time [8,10]. Tumors of this subclass show a distinct gene expression profile [18].

Currently, the only genomic multiplatform dataset that has been reported for UM is the TCGA-UVM dataset. Robertson and coworkers recently performed multi-omics on eighty UM cases within the TCGA-Project [7]. This analysis subdivided the two cytogenetic classes (di- versus monosomy of chr3) into two subclasses each, characterized by SF3B1/SRSF2 and EIF1AX mutations in association with distinct transcriptional and methylation profiles in disomic cases, and distinct gene expression and copy number alterations in monosomic cases. Data analysis has thereby been performed separately for each platform followed by a graphical presentation that reassumes the classification obtained by the analysis of data derived from the single platforms.

For the purpose of classification it appears, however, desirable to take the information from the different platforms into account simulatenously. Analytical integration of data of different platforms can be obtained by data fusion techniques that are being developed for the analysis of complex genomic data. Various approaches have been proposed [19,20] but have not yet found wide application for cancer classification despite the availability of many multiplatform datasets. A recent exhaustive overview of the application of integrative analysis on multi-level data in the field of cancers, with a focus on variable selection, can be found in [21].

Here, we applied and adapted data fusion approaches to prognostic classification of UM. We first perform a simultaneous principal component analysis (PCA), dubbed here joint Singular Value Decomposition (jSVD) but known in chemometrics as Simultaneous Component Analysis (SCA) [22] or a form of coupled matrix factorization [23]. In some works on multi-omics cancer data integration, this approach is known as the k-table method [24]. We then generalize this factorization by allowing different constraints on the factor matrices. This latter method we call joint Constrained Matrix Factorization (jCMF) and it can be computed using state of the art data fusion algorithms [25]. We compare our algorithms and their findings with a joint graph clustering, the Similarity Network Fusion (SNF) that has recently gained momentum in the field [26].

The aim of this article is the application of multiplatform data fusion techniques to the particular uveal melanoma dataset in order to verify previous classification but by using such a black box approach. We show that, in particular, the four classes described by Robertson et al. [7] can be distinguished by our methods although the main discriminator is chromosome 3 status that is associated with distinct transcriptional and methylation profiles.

## 2. Results

The TCGA-UVM dataset consists of several datasets: gene expression (mRNA and ncRNA), DNA-methylation, DNA copy number alterations (CNA) and somatic mutation data. This data has been collected for 80 patients and it has varying size. The task is to cluster these patients into different groups that predict the malignancy of the tumor. This has been done most recently in [7], where each dataset has been considered independently and then an integrative analysis has identified four clusters with increasingly worse prognosis. Ultimately, the clustering relies mostly on the CNA data.

**Figure 1:**
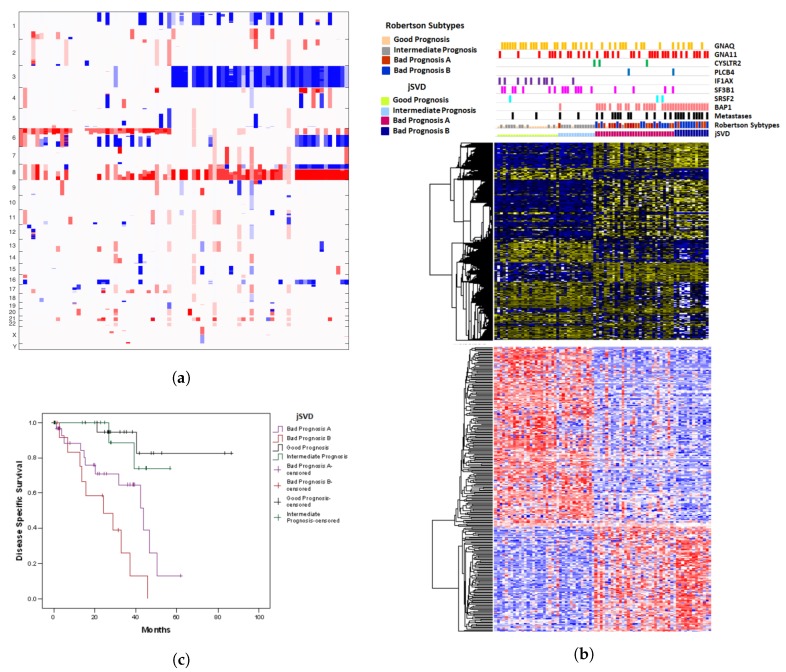
(**a**) Copy Number variation plot for the clusterings obtained by jSVD; columns = samples, rows = chromosomes. (**b**) Somatic mutation, transcriptome and methylome data for the clusterings obtained by jSVD; columns = samples, rows = genes. (**c**) Kaplan Meyer survival curves for the clusterings obtained by jSVD; *y*-axis = ratio of patients surviving, *x*-axis: time in months. Gains (red or yellow) and losses (blue) are indicated by conventional color codes.

**Figure 2:**
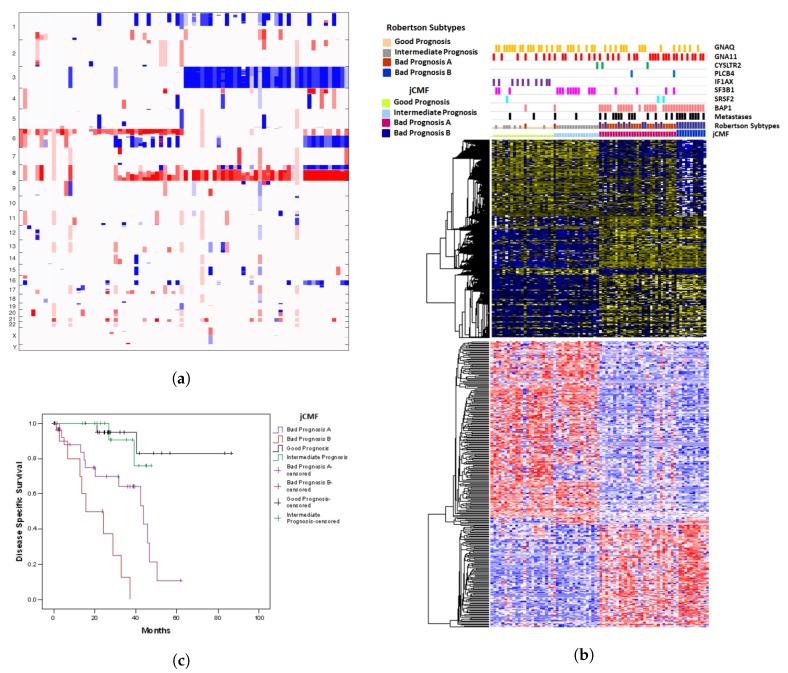
(**a**) Copy Number variation plot for the clusterings obtained by jCMF; columns = samples, rows = chromosomes. (**b**) Somatic mutation, transcriptome and methylome data for the clusterings obtained by jCMF; columns = samples, rows = genes. (**c**) Kaplan Meyer survival curves for the clusterings obtained by jCMF; *y*-axis = ratio of patients surviving, *x*-axis: time in months.

**Figure 3:**
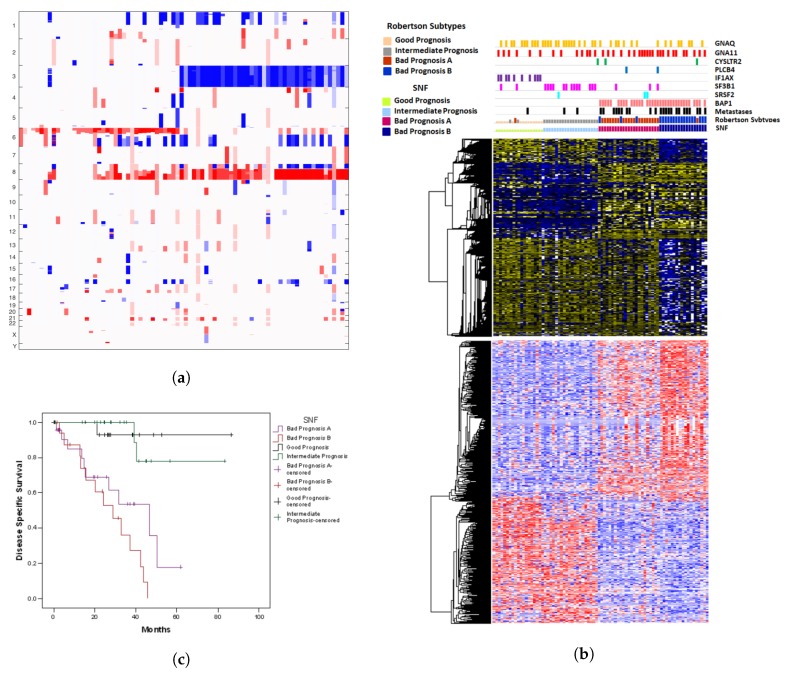
(**a**) Copy Number variation plot for the clusterings obtained by SNF; columns = samples, rows = chromosomes. (**b**) Somatic mutation, transcriptome and methylome data for the clusterings obtained by SNF; columns = samples, rows = genes. (**c**) Kaplan Meyer survival curves for the clusterings obtained by SNF; *y*-axis = ratio of patients surviving, *x*-axis: time in months.

The goal of this work is to use data fusion techniques to analyse and cluster the datasets simultaneously. This is done both to test the proposed methods on cancer data and to evaluate the validity of the former integrative analysis. In the scope of this article, we constrain ourselves to the three datasets of mRNA expression data, DNA-methylation data, and CNA data, which we will store in three matrices

$$A_{1}\in R^{80\times 20531},A_{2}\in R^{80\times 20122},A_{3}\in R^{80\times 30881}$$

respectively. The first two matrices, corresponding to expression and methylation data, are nonnegative, whereas the copy number matrix has no restrictions on the entries. See Section 4.2.1 for the preprocessing of this data, in particular for the CNA matrix. Note that we are so far unable to include the data on somatic mutations in our data fusion approach as it consists of binary data that is not only hard to handle by itself but even more difficult to “fuse” with other, less restrictive datasets. Co-mutation plots are shown in Figure 1, Figure 2 and Figure 3 but they were not used in data fusion analyses.

We propose two different algorithms that to our knowledge have so far not found application in cancer research. In the most general setting, we aim at factorizing *M* datasets $A_{i},i=1,\ldots ,M$ such that

(1) $$A_{i}\approx UV_{i}^{T}.$$

The datasets all share the same row dimension, in our case the number of patients. Therefore, the factor matrix *U* is chosen to be the same for all $A_{i}$. This means that we are trying to find a common row space that is spanned by the columns of *U*. If the dimension of this row space is small, we obtain the most important feature vectors that constitute similarities of the datasets as columns of the matrices $V_{i}$.

When the datasets $A_{i}$ consist of nonnegative information, *joint Nonnegative Matrix Factorization (jNNMF)* [27], a method that has been successfully applied in several studies [28,29,30], could be used to find the decompositions (1). In jNNMF, the factor matrices *U* and $V_{i}$ are chosen to also contain only nonnegative entries. This is done to ensure that the feature vectors in the $V_{i}$ are meaningful. If, however, one of the datasets may contain negative entries, as in our case the CNA matrix, jNNMF is not reasonably applicable, and we need to find alternative ways of joint factorization.

A first very straightforward approach to overcome this problem is to find a different constraint on the factor matrices that is more readily generalizable to different data types. Perhaps even more important in data analysis than NNMF is the *Principal Component Analysis (PCA)* that relies on the computation of a *Singular Value Decomposition (SVD)* and yields *orthogonal* feature vectors. This has the advantage that the features are complementary to each other, i.e., information found in one feature vector is strictly not found in any of the other feature vectors. Doing this simultaneously via data fusion results in a joint Singular Value Decomposition (jSVD). This is the first proposed method and described briefly in Section 4.2.2, and in more detail in [31].

On the other hand, for taking nonnegativity of some of the matrices $A_{i}$ back into account, we also propose a unified method that we call *joint Constrained Matrix Factorization (jCMF)*. Here, we decompose all matrices as in (1) but with different constraints on the feature matrices $V_{i}$, see Section 4.2.3.

To judge the justifiability of clusterings obtained from these two methods, we also test the state of the art *Similarity Network Fusion (SNF)* approach, a method that has recently gained momentum in the joint analysis of biological data, see Section 4.2.4 for more details. Here, the weighted graphs are fused together using an iterative procedure described in [26]. This ultimately yields a single graph that contains the information of all datasets $A_{i}$ and that can be clustered with standard techniques. While this method is rather heuristic, it has performed well in the past and it is widely used.

### 2.1. Results of jSVD

We first present the results obtained by the jSVD method. Figure 1a shows the copy number variations for the 80 patients ordered according to k-means clustering. We decided to set the number of clusters to four to allow for a direct comparison with the analysis of copy number alteration data that yielded four clusters [7].

Three of the four groups are easily visible: the major discriminant is chromosome 3 status, disomic (groups 1 and 2) or monosomic (groups 3 and 4). Most monosomic cases also show amplification of chr8q and a subgroup (group 4) shows deletion of chr8p. The same subgroup is distinguished by deletions of chr6q. Most disomic cases show amplification of chr6p and many of these show additional alterations, deletions or amplifications, on chr6q. The distinction of two groups within the disomic cases is less evident. Group 1 shows no deletions on chr1 that are more frequent in all the other groups. We also extracted features from somatic mutation, transcriptome and methylome data (Figure 1b) where the distinction of chr3 disomic and monosomic cases is very clear. Group 4 is distinguished by stronger methylation and gene expression of the same genes that also characterize group 3. The distinction between groups 1 and 2 is hardly visible. As expected, somatic mutations are also associated with these groups. BAP1 mutations are typical for group 3 and 4 cases, a single BAP1 mutation occurs in metastatic case of group 2. EIF1AX mutations are almost exclusively observed in group 1 whereas SF3B1 mutations show a similar frequency in groups 1 and 2. Clustering on CNA data alone, as reported by Robertson et al., yields more clearly distinguished subgroups 1 and 2, within the disomic cases, where group 1 is characterized by the absence of CNA on chr8. SVD fusion of multiplatform data by the jSVD method does not yield this distinction.

We also report on the Kaplan Meyer survival curves of the four groups in Figure 1c. As expected, groups 1 and 2 show better survival than groups 3 and 4. Survival is not significantly different between groups 1 and 2. Groups 3 shows slightly better survival than group 4.

### 2.2. A Unified Approach: jCMF

The clusterings obtained by joint SVD are satisfactory but a clear division between groups 1 and 2 is not visible. Indeed, the clustering obtained by jCMF shows a clearer division of groups 1 and 2 in the copy number variation plot, see Figure 2a, and has slightly preferable Kaplan Meyer curves, see Figure 2c. Groups 1 and 2 are distinguished by CNA on chr8.

Gene expression and methylation features again show a clear distinction between groups 1 and 2 as compared to groups 3 and 4 and between group 3 and 4 but much less so between group 1 and 2. However, jCMF groups together cases with SF3B1 mutations in group 2 (see comutation plot, Figure 2b).

### 2.3. A Comparison with SNF

This method yields almost the same clusters as discovered in the study by Robertson et al. [7]. We have included the copy number variation plot (Figure 3a), somatic mutations, gene expression features, methylation features (Figure 3b) and the Kaplan Meyer curves (Figure 3c). Again, the distinction of subgroups 1 and 2 is weak although SF3B1 mutations almost exclusively occur in group 2.

### 2.4. Prognostic Misclassification

All methods, whether based on a single data type or on data fusion, yield similar classifications (Figure 1, Figure 2 and Figure 3) yet they all misclassify at least one sample. Classification of samples from patients who did not develop metastases as “high risk” (groups 3 and 4) not necessarily indicates misclassification since a high risk tumor may respond to treatment and therefore not develop metastases, it might develop metastases beyond the follow-up period or the patient may have died before developing a metastasis. For tumors classified as “intermediate risk” (group 2) misclassification cannot be assessed since intermediate means they may or may not develop metastases in the time frame observed. On the contrary, tumors that developed metastases but are classified as “low risk” (group 1) are truly misclassified. The contingency table (Table 1) reports cases that are misclassified as “low risk” for any of the measures applied. Chr3 status, which does not allow for “intermediate risk” misclassifies 5 cases. Chi-square statistics that omit intermediate risk show the highest odds ratio for, in order, chr3 status, DNA-methylation alone, CNA and SNF, yet in the cases of CNA and SNF, these values come at the cost of many cases classified as “intermediate risk”. Data fusion techniques apparently do not reduce misclassification rates.

## 3. Discussion

Multi-platform molecular analyses have become common for many cancers and The Cancer Genome Atlas project (https://www.cancer.gov/tcga) has collected these data for thousands of tumor samples yet most analyses consider molecular data types one at a time. Data fusion techniques are being developed, amongst them matrix decomposition techniques like GSVD [32], coupled matrix factorization [23], and tensor methods as for example in [33], but they are not yet commonly applied in cancer research. In comparison, Bayesian and network-based models have been more widely applied also by biologists, see [30], in particular SNF. An exception is perhaps the jNNMF method, which is the only model based on matrix decompositions that has become somewhat state of the art in bioinformatics.

Molecular data are neither completely independent from each other nor can one data type substitute any other data type. Gene expression relies on copy number and is inversely associated with DNA-methylation but this must not be true in each single case. Therefore, it is hard to predict whether fusion of multi-platform data actually improves molecular characterization and, for example, prognostication. For the purpose to develop innovative data fusion techniques able to maximally exploit molecular data present in the TCGA database we focused on UM data since UM, though rare, is very well characterized and intrinsically simple at the molecular level as well as in terms of prognostication. Two well distinguished classes with di- and monosomy of chromosome 3 are well known since the application of cytogenetics to UM [16,34]. The analysis of somatic mutations [8] and gene expression profiles [35] further refines cytogenetics for the purpose of prognostication. Hence, any innovation will hardly do any better but it should at least reproduce what simpler methods can distinguish. For other, more complex and less well studied neoplasias, data fusion approaches might improve the distinction of molecular classes with prognostic significance.

A downside of jNNMF is that it is only applicable to a set of nonnegative matrices. In this work, it was our aim to reduce preprocessing as much as possible and since not all datasets are nonnegative, we opted to introduce two methods that can be seen as a generalization of jNNMF for other data types. We show here two data fusion approaches, jSVD and jCMF, that are characterized by a simultaneous low rank decomposition of the data matrices, thereby projecting the data on a lower dimensional shared subspace. Both are a form of coupled matrix factorization and the former is also known as Simultaneous Component Analysis (SCA) in related fields [22]. JSVD produces orthogonal classification and feature matrices while in jCMF, it is possible to adjust the data type in order to reflect the data type of the different matrices. After the projection, a simple application of the kmeans algorithm yields the different clusters. The two algorithms are compared with state of the art SNF. Just as jNNMF, our methods have some advantages over SNF: they are less heuristic and therefore less prone to overfitting, and they produce meaningful feature vectors, i.e., vectors that contain the same data type as the original dataset and can therefore be interpreted in the same fashion. However, we do not claim superiority over SNF. Similar to SNF, jSVD and jCMF reproduce the known cytogenetic classes. The distinction of subtypes within the disomic cases is less defined and might require additional information such as SF3B1 mutation status. The importance of this subclass might be underestimated using the TCGA dataset since it has limited follow-up and cases with SF3B1 mutations develop metastases several years after diagnosis of primary UM [36]. The TCGA dataset is by no means suited to develop prognostic classifiers and the prognostic power observed might strongly depend on the specific dataset. Moreover, some level of misclassification into risk classes by analyzing the primary tumor can probably not be overcome since tumor metastasization is an intrinsically probabilistic process and since disseminated tumor cells might acquire additional fitness that is not reflected by the primary tumor they are derived from [37].

We obtain different odds ratios for the different single or multi-domain methods when applied to the classification in high and low risk groups. Nevertheless, we cannot decide which of the methods tested performs best, as single misclassifications and a different number of “intermediate risk” samples can alter the odds ratio significantly. To do this, we would need independent datasets for external validation yet the TCGA data are, at present, the only source of multi-domain data. The 80 cases of the TCGA dataset are insufficient to create distinct training and validation sets. We therefore limit our interpretation to the fact that all methods tested reproduce the classification by Robertson et al. [7], which uses CNA data alone. However, a PCA-based dimensionality reduction of the CNA data alone does not yield the same clustering and more advanced algorithms are necessary.

Using UM as a test case, the present analysis shows that the new data fusion techniques work adequately. Both jSVD and jCMF are performed independently of the dataset (up to the choice of constraints in jCMF) and therefore eliminate the overfitting problem associated with heuristic procedures. Thus, they are suited for multiplatform based classification for those tumors that cannot be exhaustively classified by single platform data. In addition, as opposed to the also considered SNF, the methods yield meaningful feature vectors (more so in the case of jCMF) that can in theory be interpreted in order to gain a deeper understanding of the combination of features that determine the classifications, which is of interest to the biologist. We are working on feature extraction methods to be combined with data fusion.

## 4. Materials and Methods

### 4.1. Dataset

Multiplatform data of the TCGA-UVM collection of 80 UM (https://portal.gdc.cancer.gov/projects/TCGA-UVM) [7] were downloaded from Broad GDAC Firehose (http://gdac.broadinstitute.org/). The dataset shows several particularities: cases were selected for having either GNAQ or GNA11 mutations, double wild types were excluded, the dataset contains two unusual cases with mutations in both GNAQ and GNA11 that have never been observed in other cohorts, one case shows an unusual high mutational burden.

### 4.2. Algorithms

#### 4.2.1. Preprocessing

The matrices $A_{1}$ and $A_{2}$ for mRNA expression and gene methylation were taken directly from the TCGA-UVM dataset and not altered. They consist of nonnegative entries corresponding to the patients in the row dimension and to the genes in the column dimension. The mRNA data considers a few more genes than the methylation data and this has not been altered.

For the copy number alteration (CNA) matrix $A_{3}$ we parsed the TCGA data into the following format: Divide each chromosome into intervals of 100,000 base pairs and concatenate the chromosomes. For each patient and each interval, store the copy number data from the dataset into the corresponding entry. The data is negative if there is a deletion and positive if there is a duplication. This procedures weighs each chromosome by the number of base pairs in it and it is not biased towards chromosomes that contain more copy number variations.

We did not perform any prior variable selection, as for example discussed in [21], because the aim is to keep preprocessing at a minimum and to test the black box nature of our methods. Furthermore, it is not clear how such a removal of information before the dimensionality reduction would affect the clustering. In fact, in dimensionality reduction techniques, single features already contribute differently to the clustering according to their variance so that the influence of uninformative features is low. This equals to the effect of filtering.

#### 4.2.2. Joint SVD

Joint Singular Value Decomposition (jSVD) factorizes the datasets $A_{i}$ as

$$A_{i}^{}\approx U\Sigma_{i}^{}V_{i}^{T}$$

where the matrices *U* and $V_{i}$ have orthonormal columns and the singular value matrices $\Sigma_{i}$ are diagonal. As opposed to NNMF, there is a deterministic algorithm that computes the best approximation of a single matrix for a given subspace dimension in polynomial time. This seizes to be true for joint SVD and other methods have to be used. In this paper, we used a Riemannian trust region scheme to maximize the joint functional

(2) $$f\left(U,V_{1},\ldots ,V_{M}\right)=\sum_{i=1}^{M}{∥\mathrm{diag}\left(U^{T}A_{i}V_{i}\right)∥}^{2}$$

thus effectively maximizing the load on the diagonal [31]. It can be easily shown that this is equivalent to minimizing the sum of least squares distances

$$g\left(U,\Sigma_{1},\ldots ,\Sigma_{M},V_{1},\ldots ,V_{M}\right)=\sum_{i=1}^{M}{∥A_{i}^{}-U\Sigma_{i}^{}V_{i}^{T}∥}^{2}$$

but since the diagonal matrices $\Sigma_{i}$ are determined explicitly by $\Sigma_{i}=\mathrm{diag}\left(U^{T}A_{i}V_{i}\right)$, the problem (2) with fewer parameters is more appropriate.

The Riemannian trust region scheme yields a local minimum on the product of Stiefel manifolds. We minimize until the norm of the projected gradient is small enough, $∥\mathrm{grad}\;f\left(U,V_{1},\ldots ,V_{M}\right)∥<10^{-12}$ using the manopt toolbox for Matlab [38]. The *cluster matrix U* is then used to sort the patients into different clusters while the *feature matrices* $V_{i}$ can be used to obtain information on which genes are most impactful on the clustering. The clustering is done by simply performing Matlab’s kmeans routine 100 times and selecting the best clustering. In our experiments, subsequent runs of the Riemannian optimization yielded the same clustering each time.

#### 4.2.3. Joint Constrained Matrix Factorization

In order to maintain a reasonable interpretability, we consider different constraints on the matrices $U,V_{1},V_{2},V_{3}$. For this, we have to drop the orthogonality constraint. Instead, we constrain the matrix *U* to be column normalized $∥u_{j}∥=1$. The other matrices are constrained according to the structure of their respective dataset.

In the present case, this means that the matrices $V_{1}$ and $V_{2}$, for mRNA expression and methylation data respectively, are constrained to be nonnegative, since the expression and methylation data are nonnegative. The matrix $V_{3}$ is left without constraints because the copy number variation data can consist of both negative and positive values for deletions or duplications respectively. We use the tensorlab toolbox for Matlab in order to minimize the functional

$$h\left(U,V_{1},V_{2},V_{3}\right)=\sum_{i=1}^{3}{∥A_{i}-UV_{i}^{T}∥}^{2}$$

until the gradient is again small enough [39]. As above, the column normalized matrix *U* is used to cluster the patients with 100 iterations of the kmeans algorithm. The idea is that the different constraints on the feature matrices not only give meaningful feature vectors, but also allow for a more accurate clustering.

#### 4.2.4. Similarity Network Fusion

In recent years, a widely used algorithm for data fusion is Similarity Network Fusion [26,30]. This method relies on spectral clustering [40] where a patient similarity network is represented by a graph with edge weights given by a Gaussian kernel

$$W_{i}\left(j,k\right)=\exp\left(\frac{∥x_{j}^{i}-x_{k}^{i}{∥}^{2}}{\mu \epsilon_{j,k}^{i}}\right)$$

For a fixed matrix $A_{i}$, the $x_{j}^{i}$ represent the rows, i.e., the data for patient *j* in dataset $A_{i}$. The weight $W_{i}\left(j,k\right)$ is a measure of similarity between patients *j* and *k*. Here, μ is some parameter that can be set beforehand (our experiments worked well for μ=0.01) and $\epsilon_{j,k}^{i}$ is a normalization, see [26]. One then constructs the *graph Laplacian*

$$L_{i}=D_{i}^{-1/2}W_{i}D_{i}^{-1/2},\;D_{i}\left(j,j\right)=\sum_{k}W_{i}\left(j,k\right)$$

or a similar normalization. Calculating the lowest eigenvectors of these graph Laplacians will yield a clustering for each dataset individually [40].

In SNF, an iterative procedure fuses the individual graph Laplacians into one common matrix (which one may regard as a common graph). The same spectral clustering procedure of the obtained matrix then yields the desired classes. A downside of this method, like for all spectral clustering methods, is that it will not produce any feature vectors that correspond to the different classes, thus making the classification difficult to interpret.

#### 4.2.5. Feature Extraction

Although jSVD and jCMF do provide meaningful feature vectors, designing a procedure to evaluate these in a meaningful way was outside of the scope of this article. It would be possible to introduce an additional sparsity constraint as in [21], which can result in a clearer pronunciation of the relevant features and therefore facilitate the a posteriori feature extraction. However, it is not clear how this regularization affects the clustering, which relies on finding a common subspace for the samples. This subspace will be altered by the additional sparsity constraint. We therefore rely on state of the art techniques in feature extraction.

Group discriminant features were identified for each of the data fusion algorithms tested by multiclass significance analysis performed using the samR package [41] implemented in BioConductor [42] on transcriptome and DNA-methylation gene level data setting false discovery rate to “0”. Genes were clustered by hierarchical clustering using Euclidean distance and average linkage.

## 5. Conclusions

We have adapted data fusion techniques that have been developed for non-biological data to multi-domain cancer data using the TCGA uveal melanoma copy number alteration, DNA-methylation and transcriptome data as a test set. The algorithms have a black box nature and preprocessing is reduced to a minimum. The methods proposed provide meaningful feature vectors that hold the potential to ease biological interpretations. Molecular classification of UM, that is predominantly determined by copy number alterations, is not improved by data fusion but this is expected to be different for more heterogeneous and complex cancers. Integration of binary somatic mutation data and feature extraction methods are to be developed.

## Figures and Tables

**Table 1:** Contingency table for risk prediction.

		Observed	Predicted			n	Pearson Chi-Square *	Odds Ratio	95% Confidence Interval
			low	interm.	high				
**Robertson et al.**	**Chr3 status**	low	38	−	16	80	18.5	10.0	3.2–31.0
		high	5	−	21				
	**DNA- meth.**	low	21	10	23	69	14.0	21.9	2.7–176.5
		high	1	1	24				
	**CNA**	low	14	21	19	57	10.5	16.9	2.0–140.9
		high	1	2	23				
**Pfeffer et al.**	**jSVD**	low	21	12	21	66	11.7	11.0	2.3–52.8
		high	2	2	22				
	**jCMF**	low	21	15	18	63	13.3	12.8	2.6–62.2
		high	2	2	22				
	**SNF**	low	17	19	18	59	13.2	21.7	2.6–179.0
		high	1	2	23				

* Comparison between low and high risk, intermediate risk not considered.

## Abbreviations

The following abbreviations are used in this manuscript:

CM	cutaneous melanoma
CNA	copy number alteration
GSVD	generalized singular value decomposition
jCMF	joint constrained matrix factorization
jNNMF	joint non negative matrix factorization
jSVD	joint singular value decomposition
NNMF	non negative matrix factorization
PCA	principal component analysis
SCA	simultaneous component analysis
SNF	similarity network fusion
SVD	singular value decomposition
TCGA	the Cancer Genome Atlas
UM	uveal melanoma
